# Motor and Pulmonary Function and Mobility Disability Among Black and White Older Adults With and Without HIV

**DOI:** 10.1093/geroni/igaa057.749

**Published:** 2020-12-16

**Authors:** Brittney Lange-Maia, Aron Buchman, Sue Leurgans, Elizabeth Lynch, Melissa Lamar, Kristine Erlandson, Lisa Barnes, Brittney Lange Maia

**Affiliations:** 1 Rush University Medical Center, Chicago, Illinois, United States; 2 Rush Alzheimer’s Disease Center, Chicago, Illinois, United States; 3 University of Colorado Denver-Anschutz Medical Campus, Aurora, Colorado, United States

## Abstract

Black-White disparities in gait speed have been observed in studies of adults reporting HIV, consistent with work among older adults without HIV. However, it is unknown if racial differences exist among adults with HIV for other mobility-related factors. We aimed to determine if racial differences exist in mobility disability among older adults with and without HIV and assess if pulmonary and motor function contribute to mobility disability. We examined older adults age 50+ with HIV (N=177; 72% Black) and without HIV (N=191; 68% Black). Motor function summarized 10 motor performances including gait speed; pulmonary function summarized 3 measures assessed using hand-held spirometry. Mobility disability was based on self-report. In regression models adjusted for age, sex, medical conditions, and smoking, neither race nor HIV status were associated with mobility-related factors. However, in models stratified by HIV status, Blacks with HIV had worse motor (β=-4.3, p=0.04) and pulmonary function (β=-50.5, <0.001) and higher odds of mobility disability (odds ratio [OR]=2.9, 95% confidence interval [CI]=1.01-8.2) compared to Whites with HIV. Racial differences were not apparent among uninfected participants in motor function, pulmonary function, or mobility disability. In subsequent models, racial differences in mobility disability were attenuated and no longer significant in HIV when adjusting for motor function (OR=0.88 per/% higher motor composite, 95% CI=0.84-0.93). Racial differences in mobility disability in HIV were unaffected when controlling for pulmonary function. Results suggest that Blacks with HIV have greater mobility disability compared to Whites with HIV, and these differences are due to differences in motor function.

